# A plethora of ocular surface manifestations in a multidisciplinary ocular graft-versus-host disease unit

**DOI:** 10.1038/s41598-022-19990-z

**Published:** 2022-09-23

**Authors:** Marilia Trindade, Melina Rodrigues, Maria Eugenia Pozzebon, Francisco José Penteado Aranha, Marcos Paulo Colella, Arthur Fernandes, Denise Oliveira Fornazari, Daniel de Almeida Borges, Afonso Celso Vigorito, Monica Alves

**Affiliations:** 1 Department of Ophthalmology, School of Medical Sciences, University of Campinas (UNICAMP), Rua Vital Brasil, Cidade Universitária, 13083-888 Campinas, SP Brasil; 2 Department of Internal Medicine (Hematology), School of Medical Sciences, University of Campinas (UNICAMP), Campinas, São Paulo Brazil; 3 Department of Biosciences, University of Calgary, Calgary, Canada

**Keywords:** Haematological cancer, Corneal diseases

## Abstract

To describe the experience in a recently created ocular graft-versus-host disease unit in a tertiary hospital and to detail ocular surface features and complications after allogeneic hematopoietic stem cell transplantation (allo-HSCT). This retrospective study included all patients who underwent allo-HSCT, with or without chronic GVHD and were being monitored in the Hematopoietic Stem Cell Transplantation Unit in the UNICAMP Clinical Hospital (Campinas, Sao Paulo, Brazil) from 2015 to 2020. Patients were concomitantly evaluated by hematology and ophthalmology teams of the Ocular GVHD Unit. Hematologists performed a comprehensive systemic evaluation searching and grading mouth, skin, lungs, gastrointestinal tract, liver and genitalia GVHD. While ophthalmologists evaluated ocular symptoms through specific questionnaire (Ocular Surface Disease Index—OSDI) and a protocol of distinct ocular surface parameters for dry eye disease (1) and ocular complications, which encompassed meniscometry, non-invasive tear break-up time (NITBUT) measurement, conjunctival hyperemia quantification, meibography, fluorescein and lissamine staining and Schirmer’s test. Patients were diagnosed with chronic GVHD using the National Institutes of Health (NIH) Consensus Criteria for Chronic Graft-versus-Host Disease. The International Chronic Ocular GVHD Consensus Group (ICOGCG) score was obtained at the onset of ocular disease presentation or afterwards. A total of 82 patients underwent allo-HSCT (97.6% full matched and 2.4% haploidentical), mainly for cases of leukemia and 73.2% had chronic GVHD. Mean onset time for chronic GVHD was 232 ± 7.75 days. The mouth, skin, and eyes were the main organs involved (63%, 50%, and 48%, respectively). Symptom scores and all ocular surface parameters differ in patients with and without chronic GVHD and along different timepoints of the follow-up. Ocular complications mostly involved were severe DED and meibomian gland dysfunction, conjunctival scarring, cataract and infections resulting in keratitis and corneal perforation. As therapeutic strategies, 73% patients received preservative-free lubricants, 27% autologous serum, 48% topical steroids, 27% oral tetracycline derivatives, 22% mucolytic eye drops and 3 patients needed bandage contact lens. Ocular GVHD is a complex and challenging disease with varied manifestations, resulting in a broad range of ocular test endpoints, and inconsistent treatment responses. The main ocular presentations were dry eye, meibomian gland dysfunction and cataracts. The therapeutic approach often involves topical steroids and autologous serum tears. It is important to monitor these patients closely, so the ocular GVHD Unit may improve the care, providing prompt identification of ocular manifestations and faster treatment of complications.

## Introduction

Chronic graft-versus-host disease (cGVHD) occurs in 30% to 70% of patients who underwent allogeneic hematopoietic stem cell transplantation (allo-HSCT)^1,2^, usually with the clinical features of an autoimmune disease^3^ and the most common organs affected are skin, mouth, gastrointestinal tract, liver and eye^3^. Ocular GVHD affects 40% to 60% of allo-HSCT recipients^4^, and ocular complications have been found to significantly affect both morbidity and quality of life^5^. Ocular manifestations usually follow the involvement of other organs, such as the mouth and skin. Ocular GVHD can involve dysfunction of lacrimal gland, meibomian gland, cornea and conjunctiva^1,6^. Based on the National Institutes Health (NIH) definition, ocular findings are not sufficient alone to establish a diagnosis of chronic GVHD and it cannot be made in the absence of chronic GVHD diagnosed in another site^6,7^. The International Chronic Ocular GVHD Consensus Group (ICOGCG) has proposed diagnostic criteria for dry eye that include (2) Symptom questionnaire quantification (Ocular Surface Disease Index- OSDI), (2) Schirmer test score without anesthesia), (3) corneal fluorescein staining, and (4) conjunctival injection grade^7^ but to date we do not have definitive diagnostic criteria for ocular GVHD.

Previous studies regarding ocular GVHD showed Dry Eye Disease (1) is insufficient by itself to establish a diagnosis of chronic GVHD disease, it is one of the most common manifestations of chronic ocular GVHD and it has been considered an important complication after allo-HSCT. It occurs in 69% to 77% of cases, with typical onset being within 6 to 9 months^5,8,9^. Severe DED can progress to corneal ulceration, melting, and perforation, which, in turn, lead to serious vision impairment and restrict daily activities^10^. Effective clinical care is hindered by the overlap between normal and DED values, the lack of a gold standard test (or even an ensemble of universally accepted tests), and the insufficient agreement between the signs and symptoms of this disease^11^.

In 2015, the Ocular GVHD unit was established at in the University of Campinas (UNICAMP) Clinical Hospital and it has combined the efforts of experienced and trained ophthalmologists and hematologists. Due to the lack of diagnostic criteria for ocular GVHD, a complete routine of visual acuity, dry eye testing and systemic examination was established at the ocular GVHD unit. Ocular GVHD has been defined using both the NIH consensus criteria and the ICOGCG scoring system. Every patient follows a flowchart to evaluate endpoints of ocular signs, symptoms and complications and to direct treatment according to gravity and response to previous intervention. The goal of the ocular GVHD unit was to join ophthalmologists and hematologists working together to evaluate these patients and to create a protocol to provide pre-, peri-, and post-operative ophthalmologic care for these allo-HSCT recipients. This report describes our experience with ocular GVHD presentations and clinical outcomes in an ocular GVHD unit.

## Methods

### Study design

This retrospective study included a cohort of all patients older than 18 years-old, with malignant and nonmalignant hematological diseases who underwent a myeloablative, reduced-intensity or nonmyeloablative allo-HSTC between 2015 and 2020 at the Hematopoietic Stem Cell Transplantation Unit in the UNICAMP Clinical Hospital (Campinas, Sao Paulo, Brazil). Graft source was bone marrow or peripheral blood. All the donors were matched related or unrelated with HLA-identical or HLA-haploidentical. Conditioning regimens and GVHD prophylaxis were selected in accordance with hospital protocols. Chronic GVHD diagnosis, staging, and grading was performed by the hematologists using the 2014 Diagnosis and Staging Working Group Report from the NIH Consensus Development Project on Criteria for Clinical Trials in Chronic GVHD^12^, at the UNICAMP Clinical Hospital Ocular GVHD Unit.

A protocol was developed to assess the ocular surface and its complications. The protocol used included a set of tests such as visual acuity, non-invasive tear break-up time (NITBUT) measurement, meniscometry, conjunctival hyperemia quantification, meibography, Schirmer test and ocular surface staining tests. DED was investigated using OSDI questionnaire symptoms^12^. A complete ophthalmic and hematological evaluation was performed according to protocol with follow-up assessments 3, 6, and 12 months in all patients after transplantation or immediately when patients reported ocular symptoms after allo-HSCT, and as soon as possible when chronic GVHD was diagnosed at any site and the International Chronic Ocular GVHD Consensus Group scores were obtained at the onset of ocular symptoms^7,12^.

Of patients with available data, the age at presentation, sex, donor type and cGVHD presence were identified. The presenting of all ocular parameters and differences in severity of dysfunction as measured at the patients’ best and worst clinical presentations during the follow-up period were recorded. In addition, the presenting ocular signs and symptoms, ocular complications and treatments used were noted. In this unit, the team routinely discusses each case in order to obtain a comprehensive understanding of the natural history of GVHD and to provide an integrated evaluation of each patient’s manifestation of this complex disease.

This study was performed after approval from the local research ethics committee. It was conducted in accordance with the tenets of the Declaration of Helsinki and current legislation on clinical research. Written informed consent was obtained from all subjects after the explanation of the study procedures and requirements.

### Ocular assessment

The Ocular Surface Disease Index (OSDI) is a subjective symptom questionnaire, used as DED outcomes measurement to estimate its severity and impact. It includes 12 items concerning symptoms and visual function, impact on daily activities and environment graded from 0 (symptoms none of the time) to 4 (symptoms all the time). A score between 0 and 12 is considered normal, while a score of 13 to 22 reflects mild DED, a score of 23 to 32 represents moderate DED, and a score of 33 or above indicates severe DED. Recent DED workshops have recognized the OSDI as a valuable and reliable tool to quantify symptoms. A Portuguese language validated version was used^11^.

The ocular surface assessment included meniscometry, non-invasive tear break-up time (NITBUT) measurement, conjunctival hyperemia quantification, and meibography using the Oculus Keratograph 5 M (OCULUS Optikgerate GmbH, Wetzlar, Hesse, Germany) followed by ocular surface staining with fluorescein and lissamine, tear break-up time (TBUT) measurement, and Schirmer’s test without anesthesia. All procedures were performed by the same examiner.

Tear film stability was measured in two different ways. First, NITBUT was determined automatically using the Keratograph 5 M, as well as through the evaluation of Placido concentric rings during continuous eye-opening intervals without fluorescein stain. Next, TBUT was measured by administering 5 μl of a 2% sodium fluorescein solution (Allergan, Guarulhos, São Paulo, Brazil) and calculating the average of three consecutive break-up times determined manually using a stopwatch.

Tear volume was inferred based on tear meniscus height (TMH), which was measured in millimeters on images taken by the Keratograph 5 M.

Meibomian gland function in upper and lower lids was performed by non-contact infrared meibography using the Keratograph 5 M. Meiboscore used for each eyelid was: 0 (no loss of meibomian glands); 1 (loss of the meibomian gland involving less than one third of the total meibomian gland area); 2 (loss between one third and two thirds of the total area of ​​the meibomian gland); and 3 (loss of more than two thirds of the total meibomian gland area).

Conjunctival hyperemia was graded as, either 0 (absent), 1 (mild), 2 (moderate), or 3 (severe). Ocular surface integrity was evaluated using corneal staining, the results of which were recorded and graded on the Oxford scheme (5 corneal regions stain at a score from 0 to 3, with a total possible score of 0 to 15), and also using lissamine green staining (central nasal and temporal areas of the ocular surface at a score of 0 to 3, with a total possible score of 0 to 9).

All parameters were then collectively graded on a severity scale of 1 to 4, according to test mode. The severity of ocular surface dysfunction was classified as 1 or absent (OSDI 1–15; TFBUT 8–15; Fluorescein 0–1; Lissamine 0–1; Schirmer’s test > 10), as 2 or mild (OSDI 16–30; TFBUT 7–5; Fluorescein 2–4; Lissamine 2–3; Schirmer’s test 10–5), as 3 or moderate (OSDI 31–45; TFBUT 4–1; Fluorescein 5–9; Lissamine 4–5; Schirmer’s test 5–1), or as 4 or severe (OSDI > 45; TFBUT immediate; Fluorescein 10–15; Lissamine 6–9; Schirmer’s test 0). This numerical classification of ocular surface dysfunction has guided therapeutic decisions in clinical practice^13^.

### Statistics

Exploratory data analysis was performed through summary measures (mean, standard deviation, minimum, median, maximum, frequency, and percentage). Comparisons between groups were performed using the Wilcoxon test. P values less than 5% were considered statistically significant. Statistical analysis was performed using the Statistical Analysis System (SAS) for Windows, version 9.4 (SAS Institute Inc., Cary, North Carolina, USA).

### Ethics approval and consent to participate

Ethics approval by Research Ethics Committee of the State University of Campinas: Ethics Evaluation Submission Certificate (CAAE) No: 56897416.9.0000.5404. This study has consents to participate of all participants.

## Results

A total of 82 patients who were under a regular allo-HSCT follow-up at the Bone Marrow transplantation section at University of Campinas were included in this cohort, regardless of presence or absence of ocular symptoms despite the presence of ocular complaints. Patients’ median age was 55 years (range of 23–75 years), and 50% (n = 41) were male. Mean onset time for chronic GVHD was 232 ± 7.75 days. Almost all of the patients received related donor stem cells, while 5% received stem cells from unrelated donor. The majority of patients had an HLA-identical sibling donor (98%). Many patients (67%) received a myeloablative conditioning regimen, while 16% and 17% received a reduced intensity and nonmyeloablative conditioning regimen, respectively. Patients were classified into the groups with (n = 60) or without (n = 22) chronic GVHD (n = 82). Table 1 shows the comparison of clinical and demographic factors between the groups.

**Table 1:** Demographics data according to Chronic GVHD history.

	Without cGVHD (n = 22)	With chronic GVHD (n = 60)	Odds ratio (95% CI), p-value^†^
**Sex**			
Male	14 (63.63%)	27 (45%)	0.467 (0.147–1.419), p = 0.2122
Female	8 (36.36%)	33 (55%)	
**Underlying condition to HSCT**			
Acute myeloid leukemia	9 (40.90%)	8 (13.33%)	0.222 (0.062–0.804), **p = 0.012**
Chronic myeloid leukemia	3 (13.63%)	16 (26.66%)	2.303 (0.555–13.642), p = 0.25
Acute lymphoid leukemia	5 (22.72%)	11 (18.33%)	0.763 (0.206–3.227), p = 0.754
Chronic lymphoid leukemia	0 (0.00%)	2 (3.33%)	1.090 (0.098–65.491), p = 1.00
Myelodysplastic syndrome	1 (4.54%)	5 (8.33%)	1.909 (0.195–94.519), p = 1.00
Nonmalignant disorders	0 (0.00%)	1 (1.66%)	0.993 (0.137–34.217), p = 1.00
**Donor type**			
Related	21 (95.45%)	57 (95%)	0.905 (0.016–12.016),p = 1.0
Unrelated	1 (4.54%)	3 (5%)	
**Conditioning regimen type**			
Myeloablative	14 (63.63%)	41 (68.33%)	1.233 (0.379–3.808) p = 0.792
Reduced intensity	7 (31.81%)	6 (10%)	0.238 (0.058–0.985) **p = 0.035**
Nonmyeloablative	1 (4.54%)	13 (21.66%)	5.809 (0.762–258.367)p = 0.098
**Source of stem cells**			
Mobilized blood	18 (81.81%)	47 (78.33%)	0.803 (0.169–3.086) p = 1.0
Bone marrow	4 (18.18%)	13 (21.66%)	1.245 (0.324–5.921) p = 1.0000
Corneal disease prior to HSCT	1 (4.54%)	4 (6.66%)	1.500 (0.137–77.329) p = 1.0000
DED prior to HSCT	1 (4.54%)	4 (6.66%)	1.500 (0.137–77.329) p = 1.0000

Data expressed as mean ± standard deviation (median) or frequency.

^†^Chi-square test; Odds Ratios of presenting cGVHD regarding different demographic characteristics; HSCT: Hematopoietic stem cell transplantation; GVHD: Graft-versus-host disease; DED: Dry eye disease; 95% CI: 95% Confidence interval.

Significant values are in [bold].

The results indicate statistically significant differences and a lower chance of developing cGVHD in the group with Acute Myeloid Leukemia (OR = 0.222, 95% CI [0.062–0.804], p = 0.0120), and in the group submitted to Reduced Intensity Conditioning (OR = 0.238, 95% CI [0.058–0.985], p = 0.0352).

There is an association of cGVHD occurrence in other site with ocular involvement. According to NIH consensus criteria, there were significant differences in the odds of skin OR = 7.190, 95% CI [2.447–21.576], p = 0.0001; oral OR = 7.892, 95% CI [2.461–27.557], p = 0.0001, and liver OR = 5.786, 95% CI [2.006–17.106], p = 0.0003, with higher chance of having ocular GVHD in these groups.

### Ocular assessment after HSCT

Table 2 shows the ocular data from the patients following allo-HSCT and compares the findings according to chronic GVHD diagnosis.

**Table 2:** Ocular assessment after hematopoietic stem cell transplantation.

	Without cGVHD (n = 22)	With cGVHD (n = 60)	p-value^††^
Visual acuity	0.80 ± 0.28 (0.90)	0.65 ± 0.28 (0.85)	**0.0146**
TMH (millimeters)	0.26 ± 0.09 (0.32)	0.27 ± 0.19 (0.84)	0.8620
NITBUT (seconds)	8.36 ± 4.77 (15.63)	3.35 ± 2.91 (9.56)	**0.0191**
Hyperemia (grade 0–4)	1.04 ± 0.87 (3.00)	1.62 ± 0.93 (3.90)	**0.0090**
Fluorescein (grade 0–15)	0.54 ± 1.79 (8.00)	3.01 ± 4.15 (15.00)	**0.0090**
Lissamine (grade 0–9)	0.11 ± 0.47 (2.00)	1.10 ± 1.85 (7.00)	**0.0424**
Schirmer’s test (millimeters)	11.32 ± 5.15 (15.00)	6.39 ± 5.90 (15.00)	**0.0006**
Upper lid meiboscore (grade 0–3)	0.88 ± 0.78 (1.00)	1.89 ± 0.89 (2.00)	**0.0011**
OSDI (grade 0–100)	17.73 ± 26.63 (87.00)	44.91 ± 30.47 (100.00)	**0.0005**

Data expressed in mean ± standard deviation (median). ^†^Chi-square test; ^††^Mann–Whitney test; DED: Dry eye disease; cGVHD: Chronic Graft-versus-host disease; TMH: Tear meniscus Height; OD: right eye; OS: left eye; OSDI: Ocular Surface Disease Index.

Significant values are in [bold].

**Figure 1:**
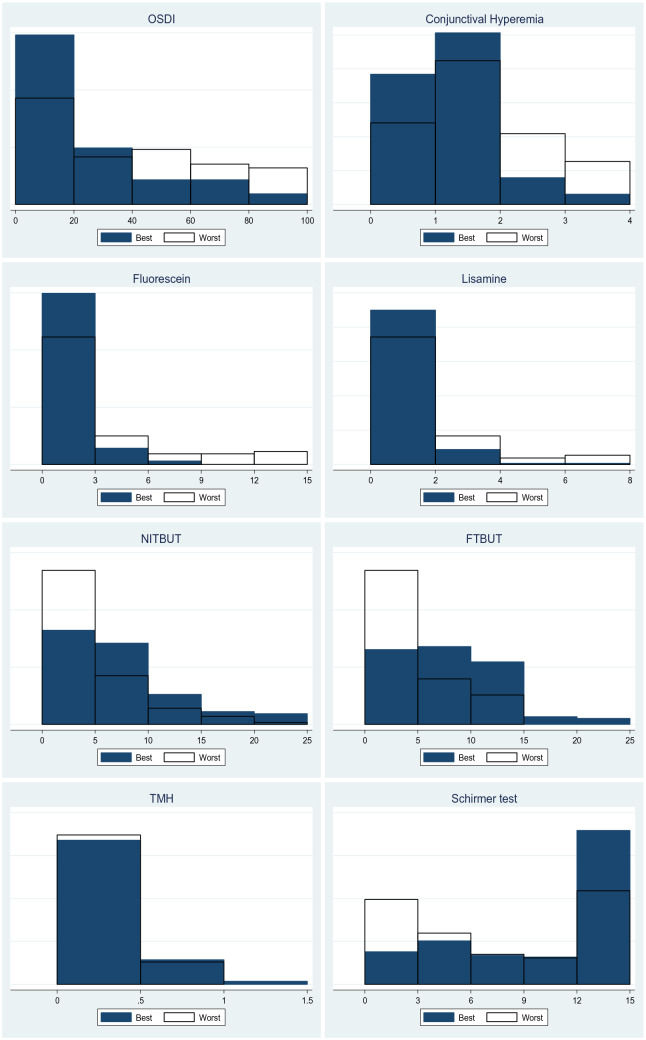
Main ocular surface parameter of patients in it best and worst clinical presentations following hematopoietic stem cell transplantation.* OSDI* Ocular Surface Disease Index;* NITBUT* Non-invasive tear break-up time;* FTBUT* Fluorescein tear break-up time;* TMH* Tear meniscus height;* OD* right eye;* OS* left eye. Of note, OSDI scores more than 40 in all patients at the worst time. TFBUT scores less than 5 in all patients at the worst time. Fluorescein scores more than 5 in most patients at the worst. Lissamine scores more than 4 in most patients at the worst. Schirmer’s test scores less than 5 in all patients at the worst time. According to severity classification, these parameters showed moderate or severe ocular surface dysfunction at the worst time in all patients after HSCT.

**Figure 2:**
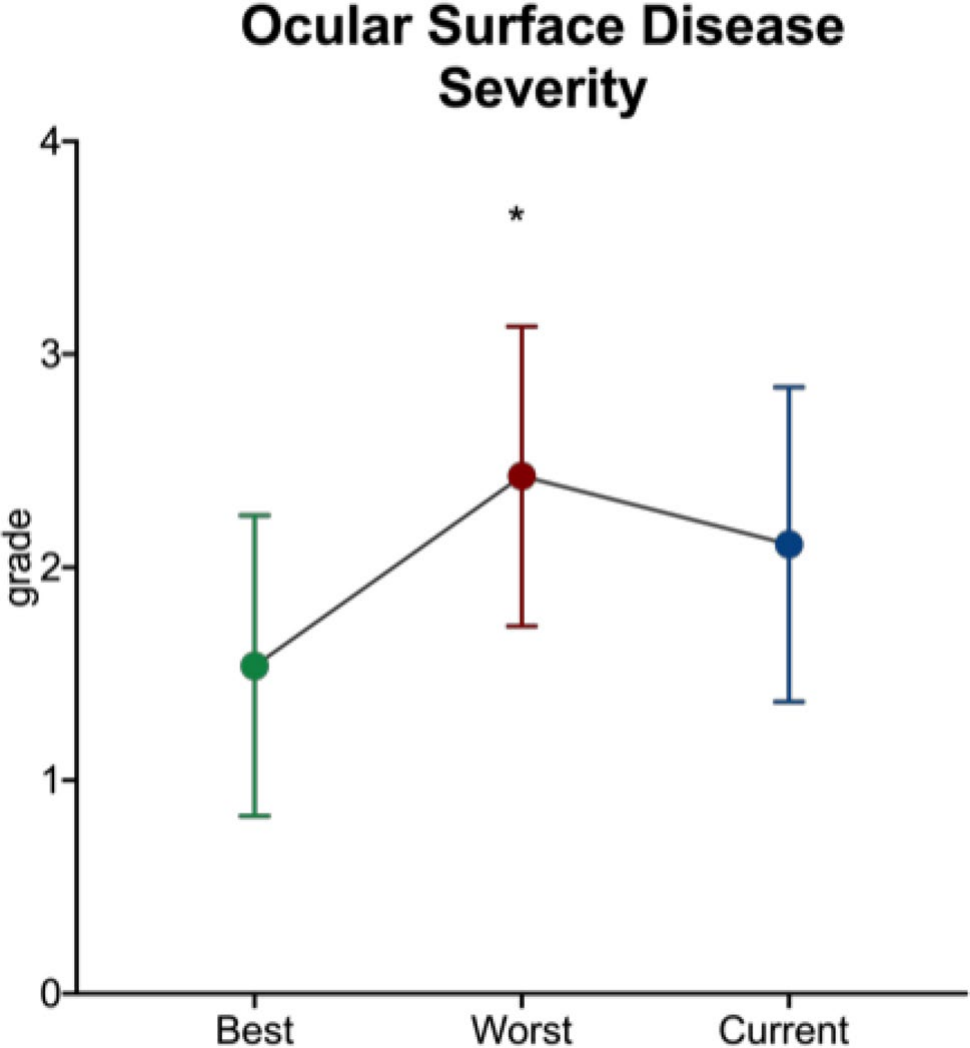
Ocular surface disease severity score based collectively on patients’ parameters at their worst, best, and current eye evaluation.

In this detailed set of ocular tests, all parameters with exception of tear meniscus high, differ in presence of chronic GVHD and are by means out of normal scores in both groups. Figure 1 displays all parameters and differences in severity of dysfunction as measured at the patients’ best and worst clinical presentations exhibited over the course of the follow-up period from 2015 to 2020. Figure 2 provides the overall scores for the severity of ocular surface dysfunction based on the clinical data, reflecting the variations in the ocular surface status during the follow-up and the impact of close treatment strategies.

### Ocular manifestations

Table 3 details the ocular manifestations among patients with and without chronic GVHD.

**Table 3:** Ocular manifestations among patients with and without graft-versus-host disease.

	Without cGVHD n = 22 (%)	With Chronic GVHD n = 60 (%)	p-value^†^
Conjunctival scarring	0 (0.00)	8 (13.33)	**0.007**
Meibomian gland dysfunction	16 (72.72)	55 (91,66)	**0.001**
Corneal opacity	0 (0.00)	4 (6.66)	**0.014**
Filamentous keratitis	0 (0.00)	6 (10.00)	**0.001**
Corneal neurotrophic ulcer	0 (0.00)	1 (1.66)	0.497
Infectious keratitis	0 (0.00)	1 (1.66)	0.541
Mucoid secretion	0 (0.00)	15 (25.00)	** < 0.001**
Corneal neovascularization	0 (0.00)	6 (10.00)	**0.001**
Corneal perforation	0 (0.00)	2 (3.33)	0.246
Cataract	4 (18.18)	17 (28.33)	**0.018**
Retinopathy	2 (9.09)	4 (6.66)	0.795
Ocular surgery	2 (9.09)	17 (28.33)	**0.009**

Data expressed as frequency of cases in the study sample. ^†^Chi-square test; GVHD: Graft-versus-host disease.

Significant values are in [bold].

Blepharitis, conjunctival scarring, leucoma, filamentous keratitis, mucoid secretion and corneal neovascularization were significantly higher among patients with chronic GVHD. Two patients had corneal ulcers (infectious and neurotrophic) that progressed to perforation. Cataract was the most prevalent ocular disease requiring surgery. Six patients had retinopathy, being two with Irvine-Gass syndrome, one had melanocytoma, one had posterior uveitis, and two developed chorioretinitis secondary to cytomegalovirus and HIV.

### Therapeutic Strategies

Patients with chronic ocular GVHD received different treatments during the study period (Table 4). Most of patients received preservative-free lubricant, almost all required topical corticosteroids (prednisolone, fluorometholone, or loteprednol) and autologous serum tears were administered to 26.66% patients. Systemic medications, as tetracycline derivatives and essential fatty acid supplements were also prescribed. Even patients without chronic GVHD needed lubricants for ocular discomfort relief.

**Table 4:** Ocular treatments.

	Without cGVHD n = 22 (%)	With Chronic GVHD n = 60 (%)	p-value^†^
Preservative-free lubricant	6 (27.27)	36 (73.33)	** < 0.001**
Lubricant with preservative	6(27.27)	11 (18.33)	0.483
Topical steroids	0 (0.00)	29 (48.33)	** < 0.001**
Mucolytic eye drop	0 (0.00)	13 (21.66)	**0.001**
Topical antibiotic	0 (0.00)	4 (6.66)	0.246
Autologous serum tears	0 (0.00)	16 (26.66)	** < 0.001**
Oral tetracycline derivatives	0 (0.00)	16 (26.66)	** < 0.001**
Essential fatty acids	2 (9.09)	19 (31.66)	**0.009**
Bandage contact lens	0 (0.00)	3 (5.00)	0.497
Penetrating keratoplasty	0 (0.00)	1 (1.66)	0.497

Data expressed as mean ± standard deviation (median) or frequency. ^†^Chi-square test; GVHD: Graft-versus-host disease.

Significant values are in [bold].

**Figure 3:**
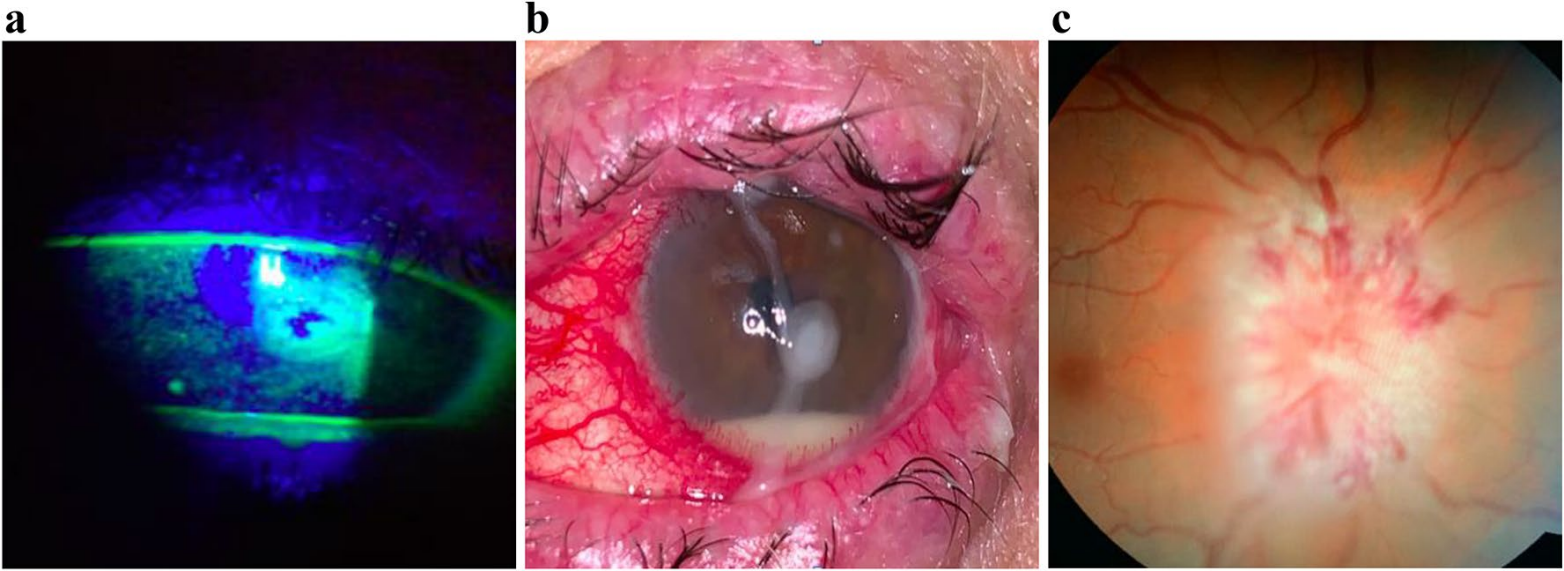
Ocular complications seen in patients treated in the ocular GVHD unit.

Figure 3 illustrates the major ocular surface complications experienced in the ocular GVHD unit. Figures 3a and 3b show a case of corneal perforation and infectious keratitis in chronic oGVHD patients, respectively. Figure 3c shows a case of leukemic optic nerve infiltration detected in a patient during the regular protocol exam prior to transplantation. Such cases demonstrate the importance of a routine and regular ocular exam as part of follow-up care for HSCT patients.

## Discussion

In this retrospective study, data from comprehensive ocular surface assessments of patients undergoing allo-HSCT were analyzed. Chronic ocular GVHD prevalence rates were similar to other studies, in which the prevalence was close to 50%^14^. Rates of chronic ocular GVHD vary widely in the literature, partly due to different diagnostic criteria. A distinct prevalence of 33% was reported in a study of 635 patients based on the 2005 NIH diagnostic criteria that included Schirmer’s test^15^. In a prospective multicenter study of patients with chronic GVHD (diagnosed according to the 2005 NIH criteria), the eyes were the third most commonly involved organ, they were affected in 51% of patients at the time of chronic GVHD diagnosis^16^. Chronic ocular GVHD is a frequent and debilitating complication of allo-HSCT that causes prolonged morbidity, affecting activities of daily living and quality of life.

Input from hematologists in the ocular GVHD unit was valuable fostering the collaboration between the ophthalmologists and HSCT physicians resulting in a faster diagnosis, more accurate identification of complications, and a prompt treatment. It represents a task force to prevent vision loss and improve patients’ quality of life.

In our study, the mouth, skin, and eyes were the most frequently involved organs among patients with systemic GVHD. The occurrence of DED, a well-known manifestation of ocular GVHD, may be multifactorial as a combined of the immune effect of HSTC, meibomian gland dysfunction (MGD), immunosuppressive therapy, total body irradiation, and ocular toxicity due to chemotherapy^17^. Systematic GVHD screening is essential for early recognition of ocular GVHD. Flowers et al.^18^ recommend routine ophthalmological screenings 3 months and at 12 months after HSCT, as well as at the time of initial chronic GVHD diagnosis at any site. Our protocol included a comprehensive ocular assessment at 3, 6, and 12 months after transplantation. Indeed, an additional assessment is recommended any time patients exhibit ocular symptoms or develop chronic GVHD at any site.

Ocular surface disease following HSCT varies in its clinical presentation, time of onset, and complications and thus requires close and intensive attention from heath care professionals. When distinct eye parameters were evaluated in patients with and without chronic GVHD, all assessments except for TMH (which reflects tear volume) were significantly altered among chronic GVHD patients. This finding might be a marker of reflex tearing, which occurs in the initial compensatory phase of the disease. Dry eye severity has also been found to worsen with time and in some specific periods such as when immunosuppressive drugs are tapered despite treatment among ocular GVHD patients. The ocular surface baseline evaluations revealed corneal involvement and MGD among the ocular GVHD patients included herein. This information, combined with the diagnostic methods reported herein, increased diagnostic performance. Pathak et al.^19^ report the sensitivity and specificity of certain diagnostic tests for ocular GVHD, such as OSDI (44% and 98%, respectively), corneal staining (91% and 54%, respectively) and TBUT (80% and 67%, respectively). A prospective study showed that reflex tearing was exhibited by 86% of patients before HSCT but began to decrease approximately 3 months after HSCT, and that mean Schirmer test values decreased to ≤ 10 mm within 6 months^20^. Conjunctival involvement in patients with chronic GVHD was characterized by hyperemia (signaling ocular surface inflammation) and cicatricial conjunctivitis as a sequela event. Recent studies found subtarsal fibrosis in several patients with chronic ocular GVHD and associated the condition with worsening corneal epitheliopathy^21,22^. The status and function of the meibomian glands among ocular GVHD patients was also considered herein. The assessments revealed mucoid secretion and blepharitis, as well as meibomian gland dropout as determined by post-operative meibography. The percentage of meibomian gland area affected has been shown to reflect the severity of ocular GVHD^22^.

Ocular GVHD is a chronic condition frequently associated with exacerbations of inflammation, ocular surface damage, DED, and vision-threatening complications such as cataract, corneal perforation and infection. Previous studies have suggested that epitheliopathy may be more severe in chronic GVHD patients when compared to other causes of DED^14,23^. In our study, the management of corneal damage with lubricants alone appeared insufficient in cases of severe epitheliopathy and abundant mucoid secretion, and both a topical anti-inflammatory (loteprednol or prednisolone acetate) and mucolytic eyedrop were required. In cases in which these additional treatments failed to control the disease, the next step was to initiate biological substitutes, such as autologous serum tears. In this study, 48% of patients required one or more therapeutic strategies in addition to artificial tears. However, despite interventions, 3% of patients continue to worsen and may develop progressive indolent corneal ulcerations that may require surgical treatment, such as keratoplasty. Fortunately, corneal ulceration leading to perforations in ocular GVHD was uncommon, as has been seen in other studies^24^. Two of the patients included herein required penetrating keratoplasty due to an infectious and neurotrophic corneal ulcer with progressive thinning and eventual perforation. Bandage contact lenses and tissue adhesive glues were applied with no improvement. Bacterial keratitis occurred due to poor surface integrity and intense immunosuppression. Cataract was the most common complication. The relevant risk factors herein included total body irradiation as part of the conditioning regimen and prolonged steroid therapy for the prevention and treatment of chronic GVHD. Inamoto et al. reported a prevalence of cataracts among allo-HSCT patients that ranged from 11 to 100%^24^. This large variability is attributable to differences in patient populations, conditioning regimens, supportive care, and length of follow up. Cataract surgery likely contributed to some of the improvement in patients’ final visual acuity when it was compared to their worst visual acuity during the study period. Among patients who have undergone HSCT, cataract may co-occur with dry eye and other manifestations of ocular GVHD. The concurrent ocular surface disease should be controlled before any surgical procedures in order to ensure better outcomes.

Some limitations of this study must be pointed out. Our sample consisted of patients that consecutively presented for consultation in different stages of the ocular GVHD follow-up. Thus, there is a lack of standardized longitudinal data to perform comparison of outcomes that would provide consistent responses about proper timeline for ocular evaluation, preventive and therapeutical strategies and impact of associated risk factors. Nevertheless, our cohort evaluation provided an overview of the variations on objective and subjective ocular parameters and some examples of severe complications occurring in distinct points of follow-up.

By comprehensively evaluating this cohort of HSCT patients we could find that although ocular surface manifestation and DED were found in both groups, with and without chronic GHVD, presentation and risk of complications were much worse in the chronic GVHD group, raising the attention for the need of clinical and ocular close evaluation and follow-up. The GVHD unit provided such approach, improving patients’ care.

## Conclusion

Chronic ocular graft-versus-host disease patients exhibit complex and chronic presentations of dry eye disease and ocular surface dysfunction that may result in ocular complications and visual impairment. The collaborative work of ophthalmology and hematology teams in the recently created ocular graft-versus-host disease unit described herein might provide early diagnosis and prompt treatment for oGVHD, optimizing patient outcomes and care.

## Data Availability

This study has data and material availability.
